# Editorial: Psychiatric insights in genetic and chromosomal disorders

**DOI:** 10.3389/fpsyt.2026.1848536

**Published:** 2026-05-13

**Authors:** Hiroki Ishiguro, Itaru Kushima, Jamie K. Capal

**Affiliations:** 1 Department of Clinical Genetics, Graduate School of Medicine, University of Yamanashi, Chuo, Yamanashi, Japan; 2 Department of Neuropsychiatry, Graduate School of Medicine, University of Yamanashi, Chuo, Yamanashi, Japan; 3 Medical Genomics Center, Nagoya University Hospital, Nagoya, Aichi, Japan; 4 Department of Psychiatry, Faculty of Medicine, Graduate School of Medicine, Nagoya University, Nagoya, Aichi, Japan; 5 Cincinnati Children’s Hospital Medical Center, University of Cincinnati College of Medicine, Cincinnati, OH, United States

**Keywords:** autism, copy number variants, Down syndrome, Fragile X syndrome, genetic counseling, neurodevelopmental disorders, respiratory sinus arrhythmia, schizophrenia

Recent work in genomic psychiatry suggests that similar neurodevelopmental and psychiatric phenotypes may arise from partly distinct mechanisms, and conversely, that shared genomic liabilities may manifest across multiple behavioral domains. A consistent message has emerged across five recent contributions: meaningful interpretation of psychiatric risk requires integration across genetic, physiological, developmental, and behavioral levels of analysis. Sleep disturbances in Down syndrome demonstrate how syndrome-specific biological vulnerability intersects with age-dependent emotional and behavioral profiles. Biobehavioral work in preschool autism and Fragile X syndrome demonstrates that the same physiological marker may have different predictive meaning across groups. Review and perspective articles on distal 1q21.1, microdeletions, and neurodevelopmental copy number variants further show how rare genomic variations can illuminate mechanisms of schizophrenia risk, eating behavior, and body mass phenotypes while exposing major gaps in phenotypic characterization. Dimensional work from genetics-first cohorts complements these findings by showing that categorical diagnoses alone do not adequately capture early clinical variability. Together, these studies support a multilevel framework in which genomic liability influences intermediate regulatory systems, which, in turn, shape heterogeneous developmental and psychiatric outcomes. Future progress will depend on longitudinal, dimensional, and genetics-first designs that improve mechanistic inference while guiding clinically useful, family-centered, and ethically informed psychiatric care.

## 1 Introduction

Neurodevelopmental and psychiatric conditions are characterized by marked heterogeneity in presentation, course, and outcome. Traditional diagnostic approaches, which rely largely on observed behavior, often group individuals with superficially similar symptoms despite important differences in the underlying biology and developmental trajectory. At the same time, genetically defined conditions may give rise to a wide range of psychiatric and behavioral manifestations that cannot be adequately captured by a categorical diagnosis alone.

These challenges have intensified interest in multilevel models linking genomic variation with intermediate physiological and developmental processes, and ultimately with clinical phenotypes. The studies highlighted in this Research Topic illustrate the value of this approach. Rather than treating behavior as a self-sufficient endpoint, they examined how psychiatric symptoms are shaped by interactions among rare genetic variations, physiological regulation, developmental timing, and syndrome-specific vulnerabilities. Methodologically, the contributions are complementary; they include empirical studies of sleep and autonomic function, a mechanistic mini-review of schizophrenia-associated microdeletions, a perspective on copy number variants and eating behavior, and dimensional work from genetic-first cohorts. Considered together, they support a framework in which genomic liability is best understood not as a direct determinant of diagnosis but as a source of probabilistic risk expressed through intermediate systems across development.

This has important clinical implications. As genomic medicine becomes more central to the assessment of genetic and chromosomal disorders, psychiatrists are increasingly asked to interpret risk in ways that are not only biologically informed, but also developmentally sensitive and psychosocially meaningful. Genetic findings can refine the diagnosis and prognosis; however, they also reshape how clinicians communicate uncertainty, support families, and plan for long-term care. Therefore, the challenge for psychiatric practice is not simply to incorporate genetic information but to do so in a way that remains person-centered, ethically grounded, and responsive to the lived experiences of patients and families.

## 2 Sleep, development, and emotional-behavioral risk in Down syndrome

Fucà et al. examined sleep disturbances in 289 children and adolescents with Down syndrome aged 6–18 years and showed that sleep problems were not uniformly distributed across development. School-aged children displayed higher rates of parasomnia-related disturbances, including disorders of arousal and sleep–wake transition problems, whereas other sleep difficulties, such as disorders of initiating and maintaining sleep, appeared to be more stable across age groups. Sleep-disordered breathing remains clinically relevant across developmental stages, consistent with the anatomical and neuromuscular vulnerabilities associated with Down syndrome.

Equally important, the psychiatric correlates of sleep disturbance differed according to developmental stage. In younger children, sleep problems were associated with somatic complaints, thought problems, and attention difficulties, whereas in adolescents, some associations broaden or shift toward internalizing features, including withdrawal or depressive symptoms. Therefore, sleep should not simply be viewed as a co-occurring medical issue in Down syndrome. Rather, it may function as an intermediate and developmentally sensitive marker linking syndrome-specific biological vulnerability to changing emotional and behavioral presentations. Clinically, this study argues for age-attuned and continuous monitoring of sleep as part of routine developmental and clinical follow-up, with integration into neuropsychiatric assessment when available.

## 3 Biobehavioral markers in autism and Fragile X syndrome

Wall and Roberts extended this multilevel perspective by examining behavioral and autonomic markers in 120 preschoolers with non-syndromic autism, Fragile X syndrome, and neurotypical development. Their study moved beyond the assumption that a biomarker has a uniform meaning across groups. Although baseline respiratory sinus arrhythmia (RSA) did not differ significantly among the three groups, its clinical significance varied. Lower RSA predicted greater autism symptom severity in non-syndromic autism, whereas elevated negative affect predicted social anxiety-related symptoms across clinical groups and was particularly informative in Fragile X syndrome.

The broader implication is that physiological and temperamental measures are not interchangeable proxies for general neurodevelopmental burden. Instead, they gain meaning in relation to the syndrome context, developmental level, and outcome domain. This is a critical issue in psychiatric research and practice. Improved diagnostic clarity will not come from simply adding biomarkers to existing categories but from understanding how specific biological signals map onto distinct symptom dimensions in different populations. Physiology does not replace behavior; it helps explain why similar behaviors may arise from different underlying regulatory processes.

## 4 Rare genomic variation as a route to mechanism in schizophrenia

In a mini-review, Guo et al. synthesized evidence linking distal 1q21.1 microdeletion to schizophrenia and illustrated how rare copy number variants can clarify the mechanisms of psychiatric risk. The deletion is reported in approximately 0.2%–0.6% of individuals with schizophrenia and is associated with an approximately eight-fold increase in risk. The authors reviewed the evidence implicating genes in the deleted region, including PRKAB2, BCL9, CHD1L, GJA5, and GJA8, in neurodevelopment, cellular signaling, chromatin remodeling, and neural communication.

The value of this contribution lies not only in summarizing a specific genetic association but also in showing how genetic psychiatry can move from locus discovery to mechanistic inference. Rather than treating schizophrenia risk variants as static predictors, this review situates them within the biological pathways that may influence neuronal differentiation, synaptic and metabolic functions, and circuit integrity. Rare variant research is especially powerful when it offers tractable models of how genomic liability may translate into intermediate dysfunction, and ultimately into clinical vulnerability.

## 5 Eating behavior, metabolism, and psychiatric genomics

Chawner’s perspective article broadens the field of view by considering eating behavior and eating disorders in individuals with rare neurodevelopmental variants. This contribution is important because it shows that psychiatric genomics cannot be confined to cognition, behavior, or psychosis; it must also address phenotypes at the interface of the brain, metabolism, and behavior. The clearest example is the 16p11.2 locus, where reciprocal copy number changes are associated with a striking mirror phenotype; deletion is linked to obesity, hyperphagia, and emotional overeating, whereas duplication is associated with underweight status, restrictive eating, and heightened satiety responsiveness.

At the same time, this study emphasizes that the field remains underdeveloped. Research has focused disproportionately on body mass index and hyperphagia, whereas more refined eating disorder phenotypes, including restrictive, avoidant, binge-related, and cross-diagnostic features, remain insufficiently characterized. By identifying these gaps, this study highlights the need for genetics-first and cross-disciplinary research that combines psychiatry, behavioral science, genetics, and metabolic medicine. Rare variants do not simply increase the risk of isolated disorders; they can reshape multiple domains of regulation and behavior in ways that challenge conventional diagnostic boundaries.

## 6 Dimensional phenotype measurement in children with rare genetic conditions

A further contribution from Chawner et al. underscores the importance of dimensional assessment in young people at high genomic risk for neuropsychiatric conditions. Studies on genetics-first cohorts suggest that clinically meaningful variability is present early in development and is often poorly captured by categorical diagnoses alone. Children carrying rare genomic variants may show uneven profiles across social communication, cognition, attention, emotional regulation, adaptive functioning, and behavior without neatly fitting into a single diagnostic box.

This perspective helps integrate other articles in the topic. If genomic liability is pleiotropic and developmentally dynamic, then categorical labels are unlikely to sufficiently account for the risk or mechanism. In contrast, dimensional approaches allow researchers and clinicians to describe how difficulties cluster within and across syndromes and to examine how these dimensions relate to physiological regulation, sleep, cognition, and specific classes of genomic variation. Thus, dimensional assessment is not merely a methodological preference; it is a necessary consequence of seriously considering genetic heterogeneity.

## 7 Discussion

Collectively, these five contributions support a coherent multilevel framework for psychiatric research on genetic and chromosomal disorders. At the first level, rare genomic variants and syndrome-specific chromosomal conditions define probabilistic, rather than fixed, outcomes. At the second level, these liabilities influence intermediate systems such as sleep regulation, autonomic function, neurodevelopmental timing, synaptic signaling, and metabolic control, which shape the expression of risk. At the third level, the resulting phenotypes appear as variable constellations of emotional, behavioral, cognitive, psychiatric, and somatic features. This model helps explain why similar diagnoses may reflect partially distinct mechanisms and why the same genomic liability may manifest differently across individuals and developmental stages.

The empirical studies by Fucà et al. and Wall and Roberts are especially useful in illustrating this principle. In Down syndrome, sleep disturbances do not simply co-occur with behavioral problems; they interact with development and may flag changing psychiatric vulnerabilities over time. In autism and Fragile X syndrome, biobehavioral markers do not function as universal correlates; instead, their predictive value depends on diagnostic and developmental context. Together, these studies show that intermediate phenotypes are the most informative when interpreted relationally, within syndromes, within ages, and with attention to the specific outcome domain being predicted.

Reviews and perspective studies have extended this logic to broader levels of psychiatric risk. Guo et al. demonstrated how a rare schizophrenia-associated microdeletion could be used to organize mechanistic hypotheses linking genes to cellular and circuit-level dysfunctions. Chawner’s perspective on eating behavior shows that psychiatric genomics must also address phenotypes traditionally divided between psychiatry and metabolic medicine. The dimensional work from genetic-first cohorts then provides a conceptual bridge: if genomic effects are pleiotropic, developmentally contingent, and distributed across multiple outcome domains, research must move beyond disorder-by-disorder models toward dimensional, longitudinal, and transdiagnostic approaches.

This framework has direct implications for clinical practice. Genetic findings can improve risk stratification and guide anticipatory monitoring; however, their value depends on how they are translated into care. Sleep screening in Down syndrome may support earlier recognition of emotional or behavioral difficulties. Genetic-first assessment may help identify children who require closer developmental follow-up, even before conventional diagnoses fully crystallize. At the same time, greater biological precision does not eliminate uncertainty; in many cases, it makes the interpretation of risks more complex. Therefore, psychiatrists need not only genomic literacy but also the ability to communicate probabilistic findings, support family understanding, and integrate biological information with developmental and psychosocial formulations.

## 8 Conclusion

The editorial discussed in this Research Topic collectively argues for a shift in the conceptualization of psychiatric phenomena in genetic and chromosomal disorders. Rather than viewing diagnosis as the primary unit of explanation, it supports a model in which genomic liability was expressed through intermediate regulatory systems and unfolded across the development into heterogeneous clinical profiles. This multilevel perspective helps to clarify both shared and distinct mechanisms across neurodevelopmental and psychiatric conditions.

Future research should prioritize longitudinal genetics-first cohorts, dimensional phenotyping, and the integration of behavioral, physiological, and genomic data. Such approaches are likely to improve mechanistic inference, refine early identification, and support individualized and developmentally informed interventions.

Within this framework, psychiatrists play a unique role as integrators, bridging genomic information, developmental context, and psychosocial meaning to support individualized and ethically grounded care. This integrative role is particularly critical in genomic medicine, where uncertainty, identity, and long-term trajectories must be addressed simultaneously.

Genomic medicine represents not only a scientific advancement that improves diagnostic precision but also a field that deeply engages with the meaning and dignity of human life. Although the current contributions provide important biological and mechanistic insights, further studies are required to directly address the psychosocial and clinical dimensions of genomic medicine in psychiatric practice. In particular, greater attention should be paid to how psychiatrists integrate diagnostic interpretations with ongoing psychosocial support across the lifespan.

